# Effects of Sad and Happy Music on Mind-Wandering and the Default Mode Network

**DOI:** 10.1038/s41598-017-14849-0

**Published:** 2017-10-31

**Authors:** Liila Taruffi, Corinna Pehrs, Stavros Skouras, Stefan Koelsch

**Affiliations:** 1 Department of Education and Psychology, Freie Universität Berlin, Berlin, Germany; 2 Department of Biological and Medical Psychology, University of Bergen, Bergen, Norway

## Abstract

Music is a ubiquitous phenomenon in human cultures, mostly due to its power to evoke and regulate emotions. However, effects of music evoking different emotional experiences such as sadness and happiness on cognition, and in particular on self-generated thought, are unknown. Here we use probe-caught thought sampling and functional magnetic resonance imaging (fMRI) to investigate the influence of sad and happy music on mind-wandering and its underlying neuronal mechanisms. In three experiments we found that sad music, compared with happy music, is associated with stronger mind-wandering (Experiments 1A and 1B) and greater centrality of the nodes of the Default Mode Network (DMN) (Experiment 2). Thus, our results demonstrate that, when listening to sad *vs*. happy music, people withdraw their attention inwards and engage in spontaneous, self-referential cognitive processes. Importantly, our results also underscore that DMN activity can be modulated as a function of sad and happy music. These findings call for a systematic investigation of the relation between music and thought, having broad implications for the use of music in education and clinical settings.

## Introduction

The ubiquity of music in human culture owes to its capability to evoke and enhance a wide range of emotions. Sadness and happiness are among the most frequent emotions evoked by music cross-culturally^1^. Sad- and happy-sounding music (henceforth referred to as sad and happy music) exist at least since antiquity, as witnessed for example from the Greek music system (6th century BC), which ascribed certain emotional qualities, including sadness and happiness, to the unique sound of musical modes.

Although over the last decade neuroscience has provided numerous insights into how sad and happy music modulate activity in brain structures involved in emotion^2^, the effects of sad and happy music on cognition remain elusive. In a previous study^3^ we found that a common use of sad (but not happy) music is to enhance self-reflection. Since the ability for self-reflection crucially requires internally-directed cognition^4^, which is typical of mind-wandering, we sought to investigate the influence of sad and happy music on mind-wandering episodes. Mind-wandering is a form of self-generated thought, which involves overcoming the constraints of the “here and now” by immersing in one’s own stream of consciousness^5^. Humans spend a substantial amount of time mind-wandering^6^, predominantly about matters of self-importance^7^, social relationships^8^, future planning^9^, and autobiographical memories^10^. Mind-wandering is associated with benefits such as facilitating creative problem solving^11^ and delaying gratification^12^, but also with costs such as disrupting ongoing task performance^13^. Research has revealed that affective processes have an important impact on spontaneous thoughts. Although there is robust evidence of an association between mind-wandering and negative affect in healthy^6^ as well as depressed individuals^14^, it has also been shown that this relationship is strongly mediated by the content of thoughts, with past-related thoughts being linked to higher levels of unhappiness^10,15^. Studies have also pointed to a key role of the qualitative features of participants’ thoughts in adaptive forms of mind-wandering. For example, thoughts focused on the future^15^ or rated as interesting^16^ lead to subsequent positive mood. Similarly, future thinking reduces cortisol levels following social stress^17^. Mind-wandering is supported by a set of brain regions typically active during rest periods, also referred to as the Default Mode Network^18–23^ (DMN). The DMN comprises most notably the medial prefrontal cortex (dorsomedial prefrontal cortex [dmPFC] and ventromedial prefrontal cortex [vmPFC]), the medial parietal cortex (posterior cingulate cortex [PCC] and precuneus [PCu]), and the lateral parietal cortex (posterior inferior parietal lobule [pIPL]). Despite a remarkable increase of research on mind-wandering^6,21^ as well as music-evoked emotions^24,25^ in recent years, it is unknown whether music with a sad and/or happy emotional tone modulates mind-wandering.

In Experiment 1A, we tested the hypothesis that sad music, compared with happy music, is associated with stronger mind-wandering. Additionally, we explored whether the qualitative content and the form of mind-wandering vary according to the music’s type (sad and happy). We analyzed a sample of 216 participants (132 female), who took part in an online probe-caught thought sampling experiment (i.e., intermittently probing individuals about their current mental state while listening to music), which combined a self-report measure of mind-wandering^20^ with an assessment of the qualitative elements and the form of self-generated thought^26–28^. Participants were asked to listen to music previously shown to evoke emotions of sadness and happiness, while keeping their eyes closed. Furthermore, they had to report their mental experience as occurring immediately before the music stopped. Each thought probe featured two initial items measuring mind-wandering. The first item (“Where was your attention just before the music stopped?”) was used as a direct self-report measure of the strength of mind-wandering (participants answered on a scale from 1 = “completely on the music” to 7 = “completely on something else”). The second item (“How aware were you of where your attention was focused?”) assessed meta-awareness as an orthogonal measure of mind-wandering (participants answered on a scale from 1 = “completely unaware” to 7 = “completely aware”). Only if participants answered > 1 on the first item, they were asked to provide further details about the content and the form of their thoughts, beginning with an open-ended question (“What were you thinking about, just before the music stopped?”). Moreover, based on previous research^26–28^, we developed eight items to assess an array of phenomenological dimensions of thought (see Table S1 for precise questions and answer scales), including (*i*) valence, (*ii*) temporal orientation (past and future), (*iii*) self-referentiality, (*iv*) social aspects (familiar and unknown people), (*v*) movements, (*vi*) bodily sensations, (*vii*) music (thinking about the musical structure and evaluating the music), and (*viii*) experiment (thinking about the experiment). The items related to the music (*vii*) were included to verify that task-relatedness was significantly lower during sad compared with happy music, in line with the process of perceptual decoupling^29^ (i.e., the disengagement of attention from perception) during mind-wandering. After these items, participants were asked whether their thoughts were based on images (“similar to a film or a painting”) or words (“similar to a dialogue or an audio-book”) to assess the form of mental activity.

Sad and happy music typically differ in tempo (with sad music featuring slower tempi than happy music, leading to lower levels of evoked arousal) and this difference, rather than the emotional quality of sad or happy music, may influence mind-wandering. For example, higher levels of sleepiness during a laboratory task were associated with increased mind-wandering^30^. Therefore, we conducted Experiment 1B (*N* = 140), which was a shorter version of Experiment 1A (see Methods) and featured slow sad and happy music stimuli (i.e., both sad and happy stimuli had the same slow tempo) as well as fast sad and happy music stimuli (i.e., both sad and happy stimuli had the same fast tempo).

Motivated by the results of Experiment 1A and given previous evidence for the DMN’s role in spontaneous cognition^18–23^, in Experiment 2 we tested the hypothesis that listening to sad compared with happy music is associated with increased DMN activity. From a sample of 24 right-handed healthy participants (12 female), we obtained whole-brain fMRI data while they listened to 4 min blocks of sad and happy music (with the same tempo) with their eyes closed. After each block, participants provided valence, arousal, sadness, and happiness ratings of their emotional state during the music. We used Eigenvector Centrality Mapping^31^ (ECM) to investigate whether sad music (compared with happy music) engages the “computational hubs” of the DMN. ECM is a graph-based network analysis technique, which measures the importance, or influence, of network nodes. ECM assigns a centrality value to each voxel (3 × 3 × 3 mm) in the brain such that a voxel receives a larger value if its time-series is strongly correlated with the time-series of many other voxels that are themselves central within the network^31^. Thus, voxels receive high eigenvector centrality values if they show functional connectivity with many other voxels that have high centrality values themselves.

## Results

### Experiment 1A

**Figure 1:**
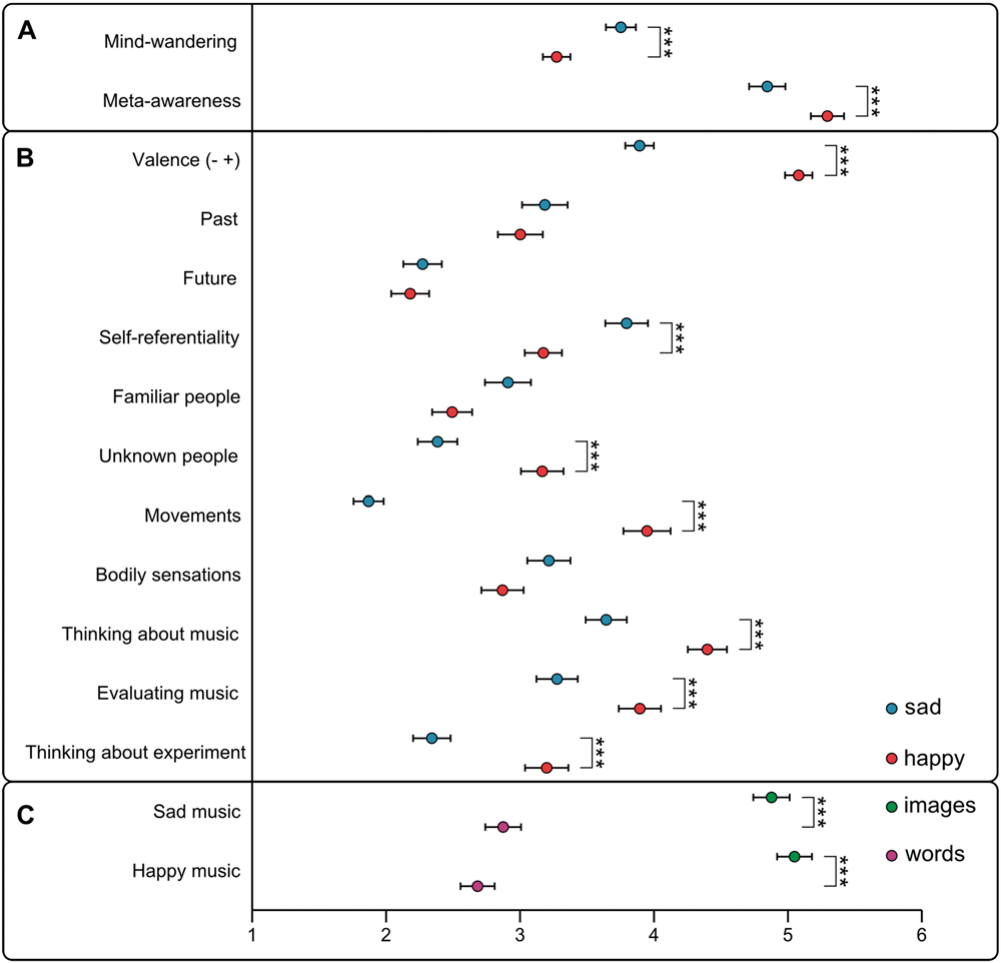
Differences in the strength of mind-wandering and its phenomenological dimensions between experimental conditions (sad and happy music). Mean ratings (± SEM) of each item featured in the thought probes are shown (answer scales 1–7; see Table S1 for precise questions). ***P ≤ 0.001, valence (− + ) = negative/positive valence. (**A**) Significantly stronger mind-wandering and significantly less meta-awareness were observed during sad (compared with happy) music (*P*-values are one-tailed). (**B**) During sad (compared with happy) music, thoughts were significantly more self-referential. By contrast, during happy (compared with sad) music, thoughts were significantly more focused on positive content, movements, unknown people, music, and experiment. (**C**) During both sad and happy music, thoughts occurred significantly more in the form of images compared with words.

**Figure 2:**
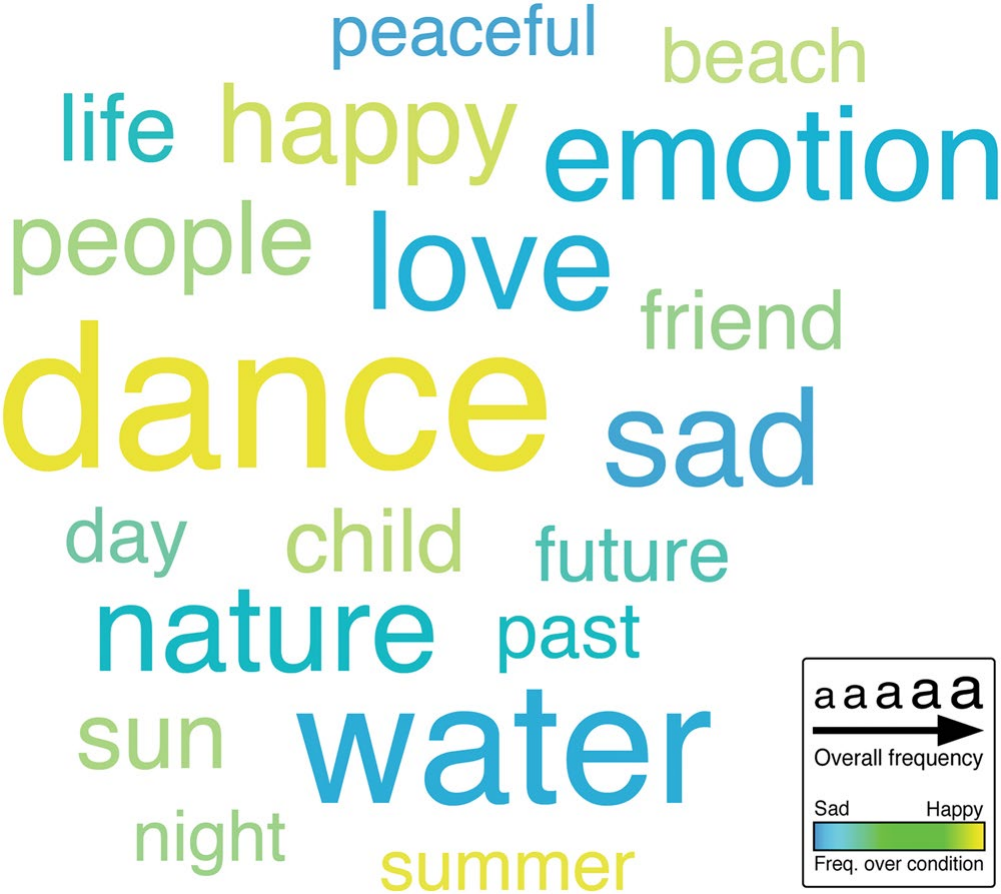
Word cloud of thought content during sad and happy music. Word size is scaled according to the overall word frequency (larger words indicate more frequent thought content over both experimental conditions). Blue color indicates thought content more frequently reported during sad music and yellow color indicates thought content more frequently reported during happy music. Thought content during sad music mainly referred to emotions and natural elements. By contrast, thought content during happy music was predominantly characterized by dance imagery.

Comparisons using paired *t*-tests (with Bonferroni-corrected *P-*values; *P*-values are two-tailed unless noted otherwise) revealed that sad music [3.71 ± 1.83 (*M* ± *SD*)] evoked significantly stronger mind-wandering than happy music (3.28 ± 1.51), *t*_(215)_ = 2.98, one-tailed *P* = 0.001, *d* = 0.21 (Fig. 1A). This was confirmed by the fact that meta-awareness, which involves one’s explicit knowledge of the current content of thoughts^29^, was significantly stronger during happy (5.25 ± 1.63) than sad music (4.86 ± 1.79), *t*_(215)_ = 3.35, one-tailed *P* < 0.001, *d* = 0.23 (Fig. 1A). Thoughts were significantly more self-referential during sad (3.80 ± 2.03) than happy music (3.17 ± 1.77), *t*_(163)_ = 3.31, *P* = 0.001, *d* = 0.26 (Fig. 1B). Conversely, thoughts were significantly more focused on movements as well as unknown people during happy [movements (3.95 ± 2.25); unknown people (3.17 ± 2.03)] than sad music [movements (1.87 ± 1.44), *t*_(163)_ = 11.31, *P* < 0.001, *d* = 0.88; unknown people (2.38 ± 1.89), *t*_(163)_ = 4.07, *P* < 0.001, *d* = 0.32; Fig. 1B], which might be due to the fact that participants imagined groups of unknown people dancing during happy music (see also Fig. 2). Overall, participants attended significantly more to happy [thinking about the music (4.40 ± 1.87); evaluating the music (3.89 ± 2.02)] than sad music [thinking about the music (3.64 ± 1.97), *t*_(163)_ = 4.72, *P* < 0.001, *d* = 0.37; evaluating the music (3.28 ± 1.97), *t*_(163)_ = 4.17, *P* < 0.001, *d* = 0.33; Fig. 1B]. The diminished attention to the music during the sad condition is in line with the decoupling of attention from external stimuli^29^, which is typical of mind-wandering. Furthermore, thoughts were significantly more focused on the experiment during happy (3.20 ± 2.07) than sad music (2.34 ± 1.79), *t*_(163)_ = 5.71, *P* < 0.001, *d* = 0.45 (Fig. 1B). Regarding temporal orientation, past- and future-oriented thoughts did not differ significantly between sad [past (3.18 ± 2.17); future (2.27 ± 1.84)] and happy music [past (3.00 ± 2.15), *t*_(163)_ = 0.84, *P* > 0.05; future (2.18 ± 1.81), *t*_(163)_ = 0.48, *P* > 0.05; Fig. 1B]. The form of mental activity (visual imagery or inner language) did not differ significantly between the two experimental conditions, and visual mental imagery was the predominant modality for both sad (4.90 ± 1.82) and happy music (5.05 ± 1.74) compared with inner language [sad music (2.90 ± 1.70), *t*_(179)_ = 9.05, *P* < 0.001, *d* = 0.67; happy music (2.70 ± 1.69), *t*_(186)_ = 11.30, *P* < 0.001, *d* = 0.83; Fig. 1C]. Thoughts were significantly more positive during happy (5.08 ± 1.31) than sad music (3.89 ± 1.36), *t*_(163)_ = 7.82, *P* < 0.001, *d* = 0.61 (Fig. 1B). Nevertheless, the analysis of the thought-reports revealed that thoughts occurring during sad music were characterized by both negative (e.g., sad) and positive (e.g., love) emotion words, indicating a mixed affective tone (Fig. 2 and Table S2).

### Experiment 1B

**Figure 3:**
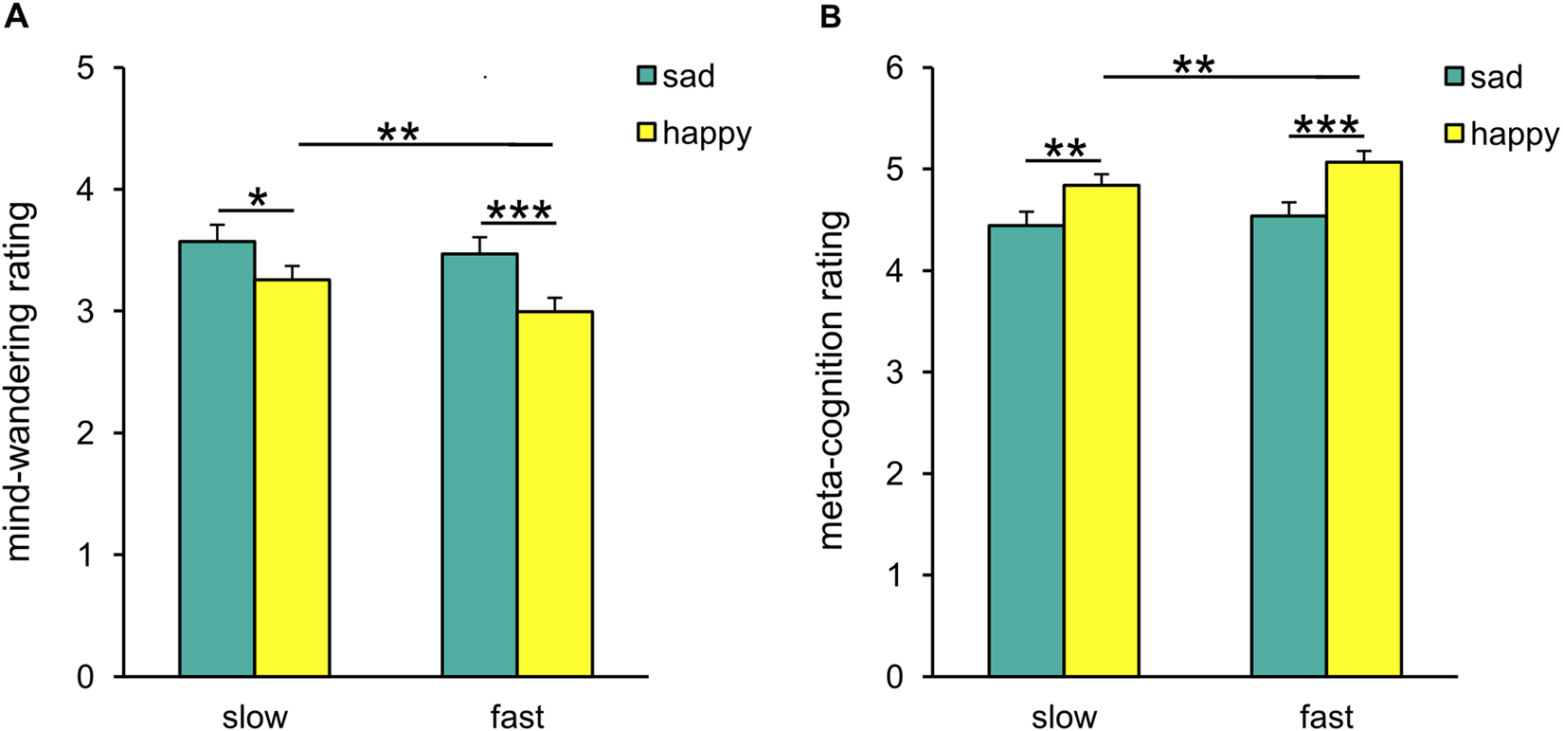
Effects of emotion (sad/happy) and tempo (slow/fast) on mind-wandering and meta-awareness. Experiment 1B replicated the results of Experiment 1A with an independent sample of participants (*N* = 140), using sad and happy music matched for numbers of beats per minute (BPM) and varying in tempo. Participants rated mind-wandering and meta-awareness on two 7-point scales (see Table S1 for precise questions). Error bars represent 1 SEM. *P < 0.05, **P < 0.01, ***P ≤ 0.001. (**A**) Significantly stronger mind-wandering was observed during sad (compared with happy) music as well as during happy slow (compared with happy fast) music. (**B**) Significantly more meta-awareness was observed during happy (compared with sad) music as well as during happy fast (compared with happy slow) music.

Two 2 × 2 repeated-measures ANOVAs with the factors emotion (sad/happy) and tempo (slow/fast) on the mind-wandering and meta-awareness ratings revealed significant main effects of emotion on both mind-wandering (*F*_(1, 139)_ = 8.56, *P* < 0.01, η_*p*_^2^ = 0.06; Fig. 3A) and meta-awareness (*F*_(1, 139)_ = 14.48, *P* < 0.001, η_*p*_^2^ = 0.09; Fig. 3B). Post-hoc paired *t*-tests (Bonferroni-corrected) showed that, for both mind-wandering and meta-awareness ratings, there were significant differences between sad slow and happy slow music (mind-wandering: *P* < 0.05; meta-awareness: *P* < 0.01) as well as between sad fast and happy fast music (mind-wandering: *P* < 0.001; meta-awareness: *P* < 0.001). Thus, confirming the results of Experiment 1A, during sad compared with happy music there was an increase in the level of mind-wandering and a decrease in the level of meta-awareness. In addition, we detected significant main effects of tempo on both mind-wandering (*F*_(1, 139)_ = 6.74, *P* = 0.01, η_*p*_^2^ = 0.05; Fig. 3A) and meta-awareness (*F*_(1, 139)_ = 7.26, *P* < 0.01, η_*p*_^2^ = 0.05; Fig. 3B). Post-hoc paired *t*-tests (Bonferroni-corrected) revealed that, for both mind-wandering and meta-awareness ratings, there were significant differences between happy slow and happy fast music (mind-wandering: *P* < 0.004; meta-awareness: *P* < 0.003), but not between sad slow and sad fast music (mind-wandering: *P* > 0.05; meta-awareness: *P* > 0.05). These results indicate that slow compared with fast music had different effects on the ratings of mind-wandering and meta-awareness depending on the type of emotion (sad, happy). Despite sad and happy stimuli being controlled for tempo, happy music evoked significantly higher arousal levels than sad music [happy slow (4.39 ± 1.09) *vs*. sad slow (2.80 ± 1.12): *t*_(139)_ = 12.61, *P* < 0.001; happy fast (5.09 ± 1.06) *vs*. sad fast (3.25 ± 1.05): *t*_(139)_ = 16.33, *P* < 0.001; happy slow *vs*. sad fast: *t*_(139)_ = 9.49, *P* < 0.001; see Table S3 for arousal ratings], indicating that other musical and/or acoustic features besides tempo contributed to arousal.

### Experiment 2

**Figure 4:**
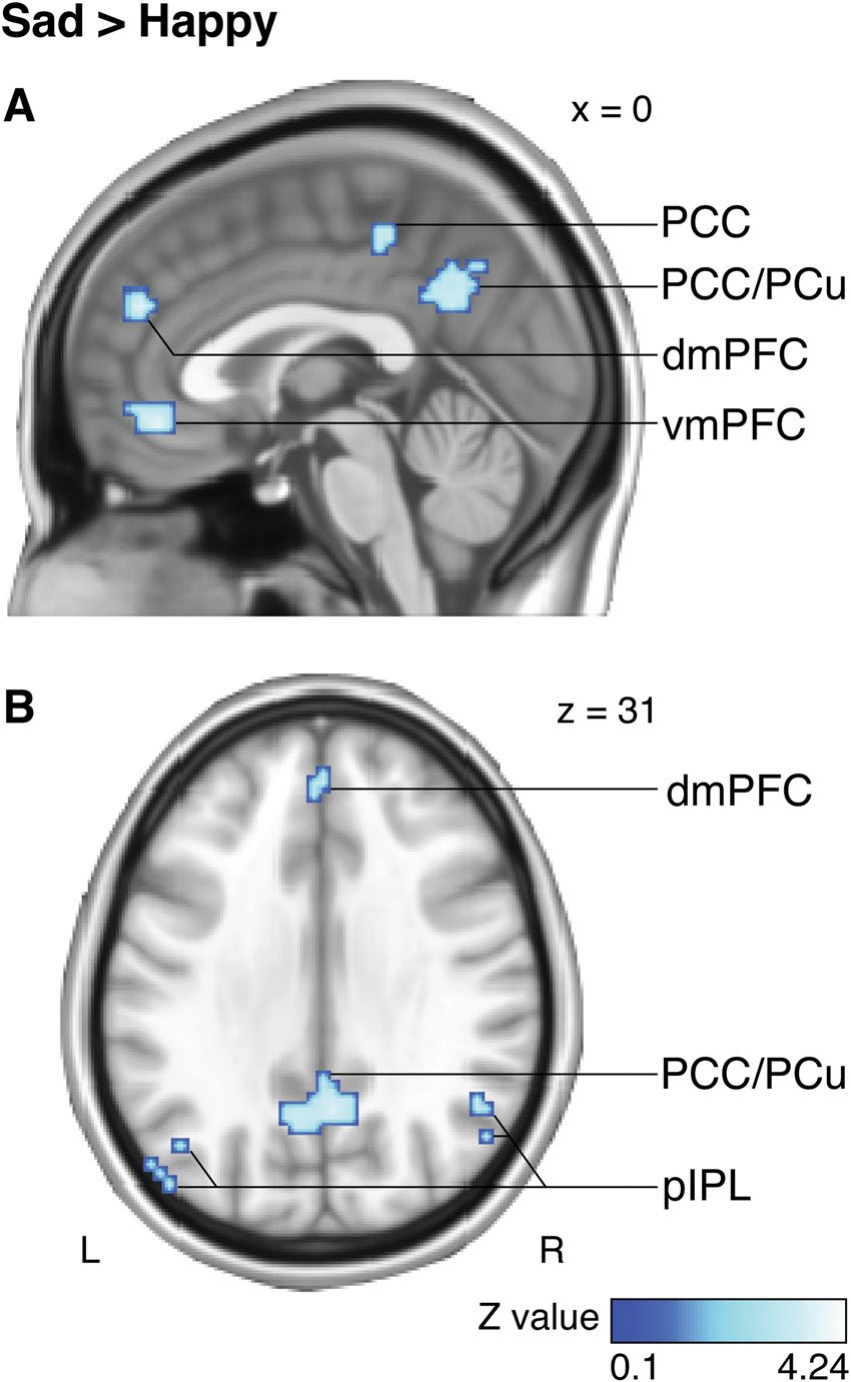
Stronger centrality of the DMN nodes during listening to sad *vs*. happy music. (**A**) Sagittal and (**B**) axial views show centrality maps obtained from a voxel-wise paired *t*-test comparing sad and happy music. Sad and happy stimuli had the same loudness and tempo (see Methods). Results were corrected for multiple comparisons (*P* < 0.05). Clusters of significantly higher centrality values were observed in the main nodes of the DMN: ventromedial prefrontal cortex (vmPFC), dorsomedial prefrontal cortex (dmPFC), posterior cingulate cortex (PCC), posterior cingulate cortex/precuneus (PCC/PCu), and posterior inferior parietal lobule bilaterally (pIPL).

Comparing centrality maps between the sad and the happy condition (*sad > happy* contrast, Fig. 4 and Table S4; for the results of the *happy > sad* contrast, see Figure S1 and Table S4) revealed a midline four-cluster pattern of significantly higher centrality values, including vmPFC (Brodmann area [BA] 32), dmPFC (BA 9), PCC (BA 23), and PCC/PCu (BAs 31 and 7). Two additional clusters were found in the pIPL bilaterally (BA 39). All of these clusters were on average within a distance of only 5 brain voxels (max. distance 6 voxels) from the brain regions of the DMN as reported in a meta-analysis^32^ on default-mode processing. Thus, in accord with the hypothesis motivated by Experiment 1A, sad music, compared with happy music, was linked to greater centrality within the core nodes of the DMN.

## Discussion

In this study we examined self-generated thought as a function of sad and happy music. Our findings reveal that music evoking sad, low-arousal emotions, compared with music evoking happy, high-arousal emotions, increased the strength of mind-wandering. Moreover, tempo can further influence mind-wandering (depending on the music’s type, sad or happy), as indicated by the observation that slow *vs*. fast happy music was associated with increased mind-wandering and decreased meta-awareness. Importantly, very little is known about which external cues trigger mind-wandering. The fact that mind-wandering can be externally modulated by means of music is in line with previous evidence^33^ of mind-wandering elicited during task such as reading aloud, and flags the importance of external emotional cues in eliciting mind-wandering episodes. The enhanced mind-wandering during sad *vs*. happy music as observed in Experiment 1 (A-B) is in line with the stronger centrality of the DMN nodes in Experiment 2, since the DMN has been indicated as the principal contributor to mind-wandering^18–23^. Consistent with our findings, a number of previous music studies^34–38^ reported activations in regions of the DMN. Specifically, the vmPFC was engaged in responses to excerpts of classical music evoking sadness^34^, and the dmPFC was engaged during listening to sad music with lyrics^35^ as well as music evoking autobiographical memories^36,37^ (past-related thoughts can often occur during mind-wandering episodes)^10^. Moreover, activation in the anterior medial frontal cortex correlated with perceived intentionality conveyed by music believed to be composed by a human *vs*. a computer^39^. Notably, our results reveal that participants’ mental activity while listening to sad *vs*. happy music was self-referential, in line with (*i*) individuals reporting to mind-wander about personally significant matters^7^ and (*ii*) evidence of a putative role of the DMN’s midline core in self-referential processing^19^. Interestingly, a recent meta-analysis^40^ showed that activity within regions of the DMN, such as mPFC, PCC and pIPL (the same areas that exhibited increased centrality in response to sad *vs*. happy music in the present study), was associated with both personal goal processing and mind-wandering. Therefore, future research could stratify the self-referential component of mind-wandering evoked in response to sad music by specifically testing whether sad music, compared with happy music, increases reflections on personal goals.

Contrary to previous findings^10^, our results do not corroborate that sad (compared with happy) mood always enhances mind-wandering in a past-oriented way. In fact, music-evoked sadness, and art-evoked sadness in general, differs in valence from “real” sadness or negative mood and represents a rather pleasurable affective state^3^ (see also Figure S2 and Table S3 for valence ratings obtained in this study). Thus, our study suggests that the multi-faceted emotional experience underlying sad music, often described by listeners as melancholic yet pleasurable^3^, shapes mind-wandering in a unique way, qualitatively non-identical to mind-wandering triggered by “everyday” negative mood. This points to a fascinating relationship between emotions evoked by artworks and thought. Likewise, we did not observe any significant difference in past-oriented thought between sad and happy music, and the analysis of participants’ thought-reports occurring during sad music reflects the mixed emotions evoked by sad music.

Sad music is slow-paced music associated with low levels of arousal, while happy music is fast-paced music associated with high levels of arousal. Because arousal is an intrinsic component of music-evoked emotions, it is challenging to disentangle its contribution to the observed relationship between sad music and increased mind-wandering (i.e., to determine to what extent spontaneous thoughts are triggered by the evoked low arousal independent of the evoked sadness). Although we controlled for the tempo of the music stimuli in Experiment 1B, happy music evoked higher arousal than sad music. This underscores that while tempo clearly influences arousal, arousal does not simply vary as a function of tempo. In spite of the difficulty to empirically separate emotion (sadness/happiness) from arousal, further research may try to pin down the mind-wandering effects on one of these two factors by using, for example, music evoking different emotions with similar arousal (e.g., sad and peaceful music, or happy and fearful music). Nevertheless, our data suggest that mind-wandering is modulated not only by arousal levels but also by the quality of the evoked emotions (sad, happy), because fast sad music, compared with slow happy music, tended to elicit *stronger* mind-wandering, despite evoking significantly *lower* arousal (Fig. 3A). Moreover, note that meditation and relaxation practices aimed at facilitating mindfulness (and at the same time avoiding mind-wandering) usually make use of music evoking low arousal emotions with peaceful and relaxed (but not sad) emotional tone. Therefore, it is unlikely that arousal is the only factor driving the changes in mind-wandering.

An additional interesting result was about the form of mental experiences during music. In particular, images (compared with words) were clearly the dominant modality for both sad and happy music, pointing to a strong link between visual mental imagery and music processing. This finding is consistent with previous studies^34,41^ reporting activations in the primary visual cortex during music listening and with the predominance of visual mental imagery during resting state^26,42^.

This study employed not only subjective but also objective indices of mind-wandering. Self-reports and neural activity were measured in separate groups of participants, assuming that mind-wandering scores for the behavioral experiments hold for the participants tested in the scanner. It will be important for future research on music and spontaneous cognition to link subjective and objective measures of mind-wandering using within-subjects designs. This would allow direct investigation of the relationship between the engagement of the DMN and the stronger mind-wandering during sad *vs*. happy music, thus strengthening overall inference. Another constraint of this study is related to the assessment of the enjoyment of the music pieces. People usually tend to mind-wander during boring and unpleasant activities^43^. Thus, the increased mind-wandering during sad music might be potentially explained by lower levels of enjoyment during sad compared with happy music. Although we did not directly measure enjoyment of the music stimuli in Experiment 1A, we collected ratings of felt valence in the corresponding pilot study (see Supplementary Information). Instead, in Experiments 1B and 2, we directly assessed felt valence in response to the music (see Table S3 and Figure S2). For all experiments (1A, 1B, and 2), valence ratings did not significantly differ between the two emotion conditions, suggesting that both sad and happy music were experienced as pleasurable, and thus enjoyable (felt valence correlates with enjoyment in the context of music)^44^. For this reason, it is unlikely that the increased mind-wandering during sad music was simply due to low levels of enjoyment of sad music. This study has specifically tested the short-term effects of sad and happy music on spontaneous cognition. It will be interesting, in future studies, to discover whether specific listening habits (e.g., regularly listening to sad and/or happy music) can affect the propensity to mind-wander in the long-term. This line of research would be highly relevant especially to music-based interventions in clinical populations (e.g., depression). Finally, the present study was conceived as a direct comparison between sad and happy music, therefore our results can not be used to infer absolute effects of sad or happy music on mind-wandering and DMN activity. Such effects should be determined, for instance, by contrasting sad and happy music with a non-music baseline condition, which discloses the average level of mind-wandering and DMN activity experienced at rest by participants in the absence of any type of music or auditory stimulation.

In conclusion, we demonstrate that music modulates self-generated thought: During sad (*vs*. happy) music, listeners direct their attention inwards, engaging in spontaneous thoughts, which are related to the self and emotional aspects of life; during happy (*vs*. sad) music, listeners are more focused on the music itself and exhibit reduced mind-wandering levels. Thus, our findings highlight the capability of music to trigger specific mental processes as a function of its emotional tone, opening a novel line of future research elucidating the impact of music on internally-oriented cognition. This has crucial implications for the application of music in a variety of domains including education and psychotherapy. The diminishing effect of happy music on mind-wandering may be beneficial for sustained attention during task performance^45^ in educational contexts, and reduce rumination as a repetitive style of thinking associated with depression^46^. The stimulating effect of sad music on mind-wandering, by contrast, could be harnessed to improve creativity^11^, social cognition^47^, and decision-making^48^ in healthy individuals. Our study also shows modulation of the DMN by music. The DMN was initially introduced as resting state phenomenon^49^ and subsequent studies revealed that its engagement reflects mind-wandering^18–23^. Our results reveal that the DMN is highly sensitive to external emotional cues conveyed by music, extending previous evidence^50^ of a link between DMN and affective processing to the music domain. Furthermore, given that aberrant DMN activity has been linked to mental disorders such as depression^51^, schizophrenia^52^, autism spectrum disorder^53^, and Alzheimer’s disease^54^, our findings provide new perspectives for the investigation of the efficacy of music therapy in the treatment of such disorders. For instance, future studies may test music’s capability to down-regulate DMN activity through the evocation of positive and highly arousing emotions, which could aid therapy in patients with a hyperactive DMN such as depressed and schizophrenic individuals^55^.

## Methods

### Experiment 1A

#### Participants

A total of 224 participants (137 female, mean age = 33.2, age range 18–55) were recruited through electronic mailing lists of students (see Supplementary Information for more details). Participants were not compensated for their participation. They completed the experiment online through a web survey platform (http://www.unipark.com/). All participants gave informed consent according to the procedures approved by the ethics committee of the Psychology Department of the Freie Universität Berlin and the experiment was performed in accordance with ethical standards outlined by the Declaration of Helsinki. Eight participants were discarded from the analysis due to low accuracy rates (≤4, see *Task design and procedure*) to follow the instructions of the experiment.

#### Music stimuli

The stimulus material consisted of four sad and four happy instrumental excerpts of film soundtracks and classical music capable of evoking sad and happy emotions (Table S5). All stimuli were unfamiliar to participants (see Supplementary Information for further details about the stimulus selection). There were four “short” (1.20–1.48 min) and four “long” (1.54–2.29 min) excerpts, counterbalanced across conditions. “Short” and “long” music stimuli were used to assess mind-wandering at different points in time after the onset of the emotion-eliciting stimulus. All stimuli were edited to have 1.5 s fade in/out ramps and were RMS-normalized (root mean square) to have the same loudness.

#### Task design and procedure

The task was designed to parallel a natural everyday setting of exposure to music, by employing unconstrained listening and use of relatively long music pieces. Participants were told that the experiment was about music, emotion, and relaxation. They were instructed to relax, listen to the music without any interruption and close their eyes (in all experiments, we opted for an eyes closed paradigm because it is typically used in resting state research^56^ and increases emotionality^57^). Moreover, they were asked to listen to the music through headphones. Participants completed a practice trial to familiarize with the task and to adjust the volume of their computer to a comfortable level. In the experimental task, the sad and happy music pieces were presented in a counterbalanced order. After each music trial, thought probes were presented. For these thought probes, participants were instructed to focus on the thoughts they had just before the music ended. At the end of the task, participants answered to an item measuring the accuracy to follow the instructions of the experiment (“How accurately did you follow the instructions of this experiment?”) on a scale from 1 (“not at all”) to 7 (“very much so”). The total length of the experiment was about 20 min.

#### Analysis of thought-reports

To gain a further insight about the content of participants’ thoughts, we examined the thought-reports provided by the participants in response to the open-ended item (Table S1) in two separate analyses.

In the first analysis, we looked at the most frequent words used by participants to describe their thoughts during sad and happy music. We used the web application Wordle (http://www.wordle.net/) to generate a word cloud summarizing our results. The word cloud was prepared in four steps. First, we excluded all the words that were reported less than a cut-off score of 10 times as well as pronouns, adverbs, articles, and prepositions (regardless of their frequency of occurrence). Words such as “music” and “thought” were also excluded from the word cloud, because they were not representative of the actual content of thoughts, but were rather biases due to the question used to inquire about participants’ mental activity (“What were you thinking about just before the music stopped?”). Second, we grouped together words with similar semantic content (e.g., happy, happiness, joyful, joy). Third, we scaled word size by their overall frequency of occurrence within reports (i.e., referring to both sad and happy conditions). Fourth, we used color to represent words’ overall frequency of occurrence over the sad and happy conditions separately.

In the second analysis, we examined participants’ thought-reports using the Linguistic Inquiry and Word Count (LIWC) software (http://liwc.wpengine.com/) to find out whether the use of positive and negative emotion words differs between thoughts that occurred during sad and happy music. LIWC identifies pre-chosen categories of language (such as positive and negative emotion) in a given text and calculates the percentage of total words that match such categories.

### Experiment 1B

#### Participants

140 participants (67 female, mean age = 31.4, age range 18–63) were recruited through electronic mailing lists of students and completed the experiment online, as in Experiment 1A. The experiment was approved by the ethics committee of the Psychology Department of the Freie Universität Berlin and was performed in accordance with ethical standards outlined by the Declaration of Helsinki. Informed consent was obtained from all participants.

#### Music stimuli

The stimulus material consisted of six sad and six happy instrumental excerpts of film soundtracks and classical music capable of evoking sad and happy emotions (Table S6). Unlike in Experiment 1A, sad and happy music stimuli were matched in pairs according to their tempo, measured in beats per minute (BPM). Each sad-happy pair had either the same BPM number or a very similar one, with a max. difference of only 4 BPM. There were six “slow” (53–88 BPM) and six “fast” (105–134 BPM) excerpts, counterbalanced across emotion conditions. The length of the stimuli ranged between 35 s and 1.24 min. All stimuli were edited to have 1.5 s fade in/out ramps and were RMS-normalized to have the same loudness.

#### Task design and procedure

The experimental protocol was the same as that of Experiment 1A, the only difference being that we assessed only mind-wandering and meta-awareness levels (both on 7-point scales), and not the form and content of spontaneous thoughts (to maintain the completion time below 15 min). After the mind-wandering and meta-awareness ratings, participants were asked to rate their emotional state during the music on four scales (valence, arousal, sadness, and happiness [7-point scales]; see Table S3 for emotion ratings). Both emotion (sad, happy) and tempo (slow, fast) conditions were presented in a counterbalanced order.

### Experiment 2

#### Participants

24 right-handed healthy participants (12 female, mean age = 25.3, age range 21–34) took part in Experiment 2 (see Supplementary Information for more details). None of these subjects participated in Experiment 1 (A-B). Participants either received course credit or 10€/h for participation. All participants gave written informed consent. The experiment was approved by the ethics committee of the Psychology Department of the Freie Universität Berlin and was performed in accordance with ethical standards outlined by the Declaration of Helsinki.

#### Music stimuli

The stimulus material included four sad and four happy instrumental excerpts of film soundtracks capable of evoking sad and happy emotions (Table S7). There were four “short” (35–37 s) and four “long” (1.18–1.30 min) excerpts, counterbalanced across conditions. We controlled sad and happy stimuli for differences in tempo characteristics: first, we matched the stimuli into sad-happy pairs according to their tempo (for each pair, sad and happy stimuli had the same or a very similar number of BPM). Then, for each sad-happy pair, we generated an isochronous sequenced electronic beat track and overlaid both the sad and the happy excerpts from that pair with that beat track. Slight tempo variations that led to deviations from the beat track were corrected using the time stretching and fit to tempo functions in FL Studio (https://www.image-line.com/flstudio/). Thus, both sad and happy excerpts from each pair were overlaid with an identical beat track, leading to the same perceived tempo and similar vestibular responses for sad and happy music (see Supplementary Information for more details about the stimulus preparation and selection). All excerpts were edited to have 1.5 s fade in/out ramps and were RMS-normalized to have the same loudness. Stimuli of the same emotion category were concatenated into blocks of 4 min duration (no stimulus was repeated), resulting in one 4 min stimulus block per experimental condition. The use of only one 4 min block of music per condition ensured optimal data for the application of the fMRI data analysis, in which we adopted Eigenvector Centrality Mapping^31^ (ECM). ECM requires relatively long trial periods, but has the advantage that only one trial per condition is sufficient per subject.

#### Task design and procedure

Prior to the fMRI measurements, participants were tested on their familiarity with the selected music pieces (see Supplementary Information for further details). In the scanner participants were presented with the sad and happy excerpts. We also presented blocks with dissonant sad and dissonant happy music, as well as with neutral music (results involving the neutral stimuli are reported in the Supplementary Information). The order of blocks was pseudo-randomized across subjects. Stimuli were presented via MRI-compatible headphones (under which participants wore earplugs) at a comfortable volume level, using Presentation (https://www.neurobs.com/). Participants were instructed to close their eyes and relax during the music listening. Each block of music stimuli was followed by a 2 s signal tone, signaling to participants to open their eyes, and then by a 16 s evaluation period, during which participants were asked to indicate their overall emotional state during the music listening, using a response pad they held in their right hands. Ratings about felt emotions were obtained on four scales (valence, arousal, sadness, and happiness [6-point scales]; Figure S2). The rating period was followed by a silence period of 10 s to avoid emotional blending between different blocks of stimuli. The total length of the experiment was about 27 min.

#### fMRI data acquisition

MRI data were acquired using a 3.0 T MRI scanner (Magnetom TIM Trio, Siemens, Erlangen, Germany) at the Dahlem Institute for Neuroimaging of Emotion. Prior to functional scanning, a high-resolution (1 × 1 × 1 mm) T1-weighted anatomical reference image was obtained from each participant using a rapid acquisition gradient echo (MP-RAGE) sequence. For the functional session, a continuous echo planar imaging (EPI) sequence was used (37 slices interleaved; slice thickness = 3 mm; interslice gap = 0.6 mm; TE = 30 ms; TR = 2250 ms; flip angle = 70°; matrix = 64 × 64; FOV = 192 × 192 mm). To minimize susceptibility artifacts in areas such as the orbitofrontal cortex and the temporal lobes, the acquisition window was tilted at an angle of 30° to the intercommissural (AC-PC) plane^58,59^.

#### fMRI data processing

Functional MRI data were processed using the software LIPSIA 2.1 (http://www.cbs.mpg.de/institute/software/lipsia/). Prior to statistical analysis, functional images were corrected for slicetime acquisition and normalized into MNI-space-registered images with isotropic voxels of 3 mm³. Low frequency drifts in the fMRI time-series were removed using a high-pass filter with a cutoff frequency of 1/90 Hz and functional images were spatially smoothed using a Gaussian kernel of 6 mm full-width at half-maximum. Furthermore, the mean signal value per scanned volume was computed and regressed out of each participant’s data. The movement parameters of each participant were also regressed out of the respective fMRI time-series to control for motion artifacts.

#### ECM analysis

ECM analysis was carried out in two steps. On the first level, whole-brain eigenvector centrality maps were computed separately for each participant during each 4 min experimental condition. On the second level, eigenvector centrality maps were compared between the two experimental conditions using voxel-wise paired *t*-tests. Results were corrected for multiple comparisons using cluster-size and cluster-value thresholds obtained by Monte Carlo simulations^60^ with a significance level of *P* < 0.05.

### Data Availability

The datasets analyzed in the current study are available from the corresponding author on reasonable request.

## Electronic supplementary material

Supplementary Information
