# Supplementary material for: Effects of Sad and Happy Music on Mind-Wandering and the Default Mode Network
Source: Sci Rep. 2017 Oct 31;7:14396. doi: 10.1038/s41598-017-14849-0 (PMC5663956; doi:10.1038/s41598-017-14849-0)
Supplement: Supplementary file 1 — Supplementary Information [file 41598_2017_14849_MOESM1_ESM.pdf]

Supplementary Information for

Effects of Sad and Happy Music on Mind-Wandering and the Default Mode Network

Liila Taruffi[1], Corinna Pehrs[1], Stavros Skouras[1], and Stefan Koelsch[2]

[1]Department of Educational Sciences and Psychology, Freie Universität Berlin

[2]Department of Biological and Medical Psychology, University of Bergen

Corresponding author:

Liila Taruffi
Dept. of Educational Sciences and Psychology, Freie Universität Berlin
Habelschwerdter Allee 45, 14195 Berlin, Germany
Tel. +49 3083857537
liilataruffi@zedat.fu-berlin.de

Supplementary Methods

Experiment 1A

Participants details. 79% of the participants were European, 8.9% North American, 8.5% Asian, 2.7% Australian, and 0.9% South American. 46% of the participants reported to be non-musicians (never played an instrument), 33.9% amateur musicians (never received musical training), 11.2% semi-professional musicians (received musical training, but music is not main profession), and 8.9% professional musicians (playing music as main profession).

Stimulus selection. Music stimuli were carefully selected to ensure their capability to elicit sadness and happiness, while avoiding familiar pieces to reduce potential biases due to memory effects (Pereira *et al.*, 2011). This was proven by the successful use of the stimuli in a previous study (Pehrs *et al.*, 2014) and a behavioral pilot experiment. In the pilot experiment, 30 volunteers (17 female, mean age = 32.1, age range 18-53) listened to 12 sad and 12 happy pieces, presented in a counterbalanced order, and were then asked to rate their emotional state during the music as well as their familiarity with each piece on five scales (valence, arousal, sadness, and happiness [7-point scales]; familiarity [4-point scale]). Based on the pilot ratings, we chose the most homogeneous set of sad and happy stimuli, with the highest significant differences to the opposite affective tone, in order to assure orthogonality of emotional experimental conditions (P-values for all emotion dimensions $< .001$ except for valence, Bonferroni-corrected). Thus, for the sad condition, the selected set of stimuli was rated as highly pleasant [5.07 ± 1.23 ($M \pm SD$)], slightly arousing (2.40 ± 0.91), clearly sad (4.40 ± 1.39), not very

happy (2.33 ± 0.79), and unfamiliar (1.29 ± 0.52). For the happy condition, the selected set of stimuli was rated as highly pleasant (5.23 ± 1.12), very arousing (4.92 ± 1.26), clearly happy (5.17 ± 1.26), not sad at all (1.34 ± 0.46), and unfamiliar (1.36 ± 0.45).

Experiment 2

Participants details. Participants were screened for depressive symptoms, alexithymia, and sensitivity to music reward, using respectively the Quick Inventory of Depressive Symptomatology (QIDS-SR; Rush *et al.*, 2003), the Toronto Alexithymia Scale (TAS-20; Bagby *et al.*, 1994), and the Barcelona Music Reward Questionnaire (BMRQ; Mas-Herrero *et al.*, 2013). All participants scored below 6 on the QIDS-SR and 52 on the TAS-20 (thus, none of the participants were depressive or alexithymic). With regard to the BMRQ, all participants scored between 40 and 60 on the two factors of *emotion evocation* and *mood regulation*, indicating an average sensitivity to reward derived from music-evoked emotional experiences. All participants were German native speakers. None of the participants were professional musicians. 58.3% of the participants were non-musicians, 29.2% amateur musicians, and 12.5% semi-professional musicians. Participants' favorite musical genres fell into the following categories: 25.7% rock, 20% electronic, 15.7% pop, 15.7% classical & soundtrack, 12.8% jazz, 5.7% reggae, and 4.4.% other. Exclusion criteria were prior history of major neurological or psychiatric disorder, alcohol and other drug abuse, and excessive consumption of alcohol or caffeine as well as poor sleep during the 24 hours before the experimental session.

Stimulus preparation and selection. We initially selected a large number of

instrumental excerpts of sad and happy film soundtracks, considered capable of evoking emotions of sadness and happiness, respectively. We avoided popular music themes to control for memory effects (similarly as in Experiments 1A and 1B) and we matched sad and happy pieces in instrumentation. Importantly, because the tempo, measured in beats per minute (BPM), is usually faster for happy than sad music, and because music beats also evoke vestibular responses (potentially leading to the activation of vestibular cortical areas, which overlap in part with areas implicated in emotional processing; Koelsch, 2014), we ensured that both sad and happy excerpts had the same tempo. To achieve this, we compiled sad and happy pairs of excerpts, featuring the same or a very similar number of BPM, and we combined them with an isochronous sequenced electronic beat (as in Pehrs *et al.*, 2014), which contained sounds of drum kits or percussions and made the tempo clear and noticeable to participants. We composed and added such electronic beat to each pair of excerpts using the software FL Studio (https://www.image-line.com/flstudio/). Different types of beats were used, following the rule of applying the same beat to each pair (sad, happy) of excerpts. The volume of the beats was set to 6 dB below the volume of the original music excerpts during the rendering of the music stimuli. A behavioral pilot study was then performed to determine the best pairs (sad, happy) of stimuli capable of evoking emotions of sadness and happiness. 42 volunteers (24 female, mean age = 28.1, age range 18-38) listened to 15 sad and 15 happy pieces, presented in a counterbalanced order, and were then asked to rate their emotional state during the music on four scales (valence, arousal, sadness, and happiness [7-point scales]). Based on the emotion ratings, we selected the four “sad-happy” pairs of excerpts that were most consistently identified as belonging to

their respective emotion category.

Neutral stimuli. In the scanner participants were also presented with neutral stimuli. These were isochronous tone sequences for which the pitch classes were randomly selected from the pentatonic scale. They featured the same beat track of the corresponding sad-happy stimulus pair (thus, neutral and emotion stimuli had identical numbers of BPM). They were generated using the MIDI (Musical Instrument Digital Interface) toolbox for Matlab (Eerola and Toiviainen, 2004) and edited to have the same length, timbre, fade in/out ramps, as well as loudness of the corresponding sad-happy excerpts.

Controlling for familiarity. Participants listened to short excerpts (15 s) of the selected sad and happy stimuli and indicated their familiarity with each excerpt on a scale ranging from 1 (“I have never heard this piece before”) to 5 (“I know this piece”). Participants were not included in the fMRI session if they were familiar with any of the music pieces. A paired *t*-test showed that there was no significant difference in familiarity between the happy (1.62 ± 0.57) and the sad pieces (1.57 ± 0.63), $P > .05$. A minimum of 14 days passed between this behavioral session and the fMRI experiment to avoid memory effects (Pereira *et al.*, 2011).

Supplementary Analyses

Experiment 2

Behavioral ratings for neutral stimuli. Participants rated their emotional state on four

6-point scales (sadness, happiness, valence, and arousal). Neutral stimuli (2.83 ± 1.01) were rated significantly less sad than sad stimuli (4.54 ± 0.83), $P < .001$ (Fig. S2C). Similarly, neutral stimuli (2.46 ± 1.02) were rated significantly less happy than happy stimuli (5.42 ± 0.72), $P < .001$ (Fig. S2D). Moreover, neutral stimuli (3.00 ± 1.32) were rated significantly less pleasant than sad (4.58 ± 1.10) and happy stimuli (5.21 ± 0.88), all *Ps* < .001 (Fig. S2A). Finally, arousal ratings did not differ significantly between neutral (2.79 ± 0.88) and sad stimuli (3.21 ± 1.18), $P > .05$ (Fig. S2B); however, happy stimuli (3.75 ± 0.90) were rated significantly more arousing than neutral stimuli, $P < .001$ (Fig. S2B).

Eigenvector centrality mapping (ECM) comparisons with neutral stimuli (ROI analysis). To evaluate how stimuli matched for tempo but without a sad or a happy emotional tone modulate DMN activity in comparison with the emotion conditions, we compared ECM values in the nodes of the DMN (vmPFC, dmPFC, PCC, PCC/PCu, and pIPL bilaterally) between sad and neutral stimuli, as well as between happy and neutral stimuli, using ROI analyses. ROI analyses were conducted for the significant clusters identified in the main ECM analysis comparing sad with happy music (see main text and Table S4). Paired *t*-tests for these ROIs were carried out for the contrasts happy *vs.* neutral and sad *vs.* neutral. Significantly higher centrality values within the six DMN nodes were observed during neutral compared with happy music stimuli (all *Ps* < .05). Significantly higher centrality values were found in the vmPFC during sad compared with neutral stimuli ($P = .03$), and all the remaining DMN structures showed slightly higher centrality values for the contrast sad *vs.* neutral (although these differences were

statistically not significant; all $Ps \approx .1$), pointing to a trend towards increased DMN activity during sad *vs.* neutral music (note that the likelihood that ECM values are higher for the neutral than for the sad condition in all six ROIs is 1/64). Thus, the results suggest that, compared with neutral stimuli, happy music reduces DMN activity, while sad music increases DMN activity. Although the evidence that sad (compared with neutral) music enhances DMN activity is only trend-wise significant, note that the neutral stimuli were more similar to the sad than the happy excerpts with regard to the evoked arousal (arousal ratings did not significantly differ between sad and neutral stimuli, $P > .05$, but significantly differed between happy and neutral stimuli, $P < .001$; see *Behavioral ratings for neutral stimuli*). Moreover, because the neutral stimuli were experienced as less pleasurable compared with the sad and happy ones (both $Ps < .001$; see *Behavioral ratings for neutral stimuli*), they may have enhanced mind-wandering levels (mind-wandering increases during boring and unpleasant activities; see Kane *et al.*, 2007 and main text on p. 12). For these reasons, the results of the ROI analyses should be verified by future studies employing neutral stimuli that are better controlled for arousal and valence. This could be done, for example, through the use of more ecologically valid neutral stimuli such as “real” music evoking neither sadness nor happiness.

Supplementary Figures

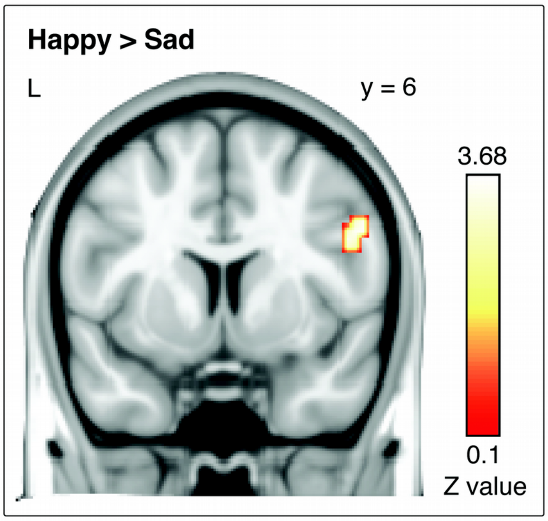

Figure S1. Increased centrality in the right inferior frontal gyrus (IFG) during listening to happy *vs.* sad music. Shown is a cluster of significantly higher centrality values located in the right IFG (pars opercularis) extending into the right precentral gyrus. The pars opercularis of the IFG (BA 44) is implicated in the processing of music syntax (Maess *et al.*, 2001), consistent with the results of Experiment 1A, indicating more focus on the musical structure during happy (compared with sad) music (Fig. 1B).

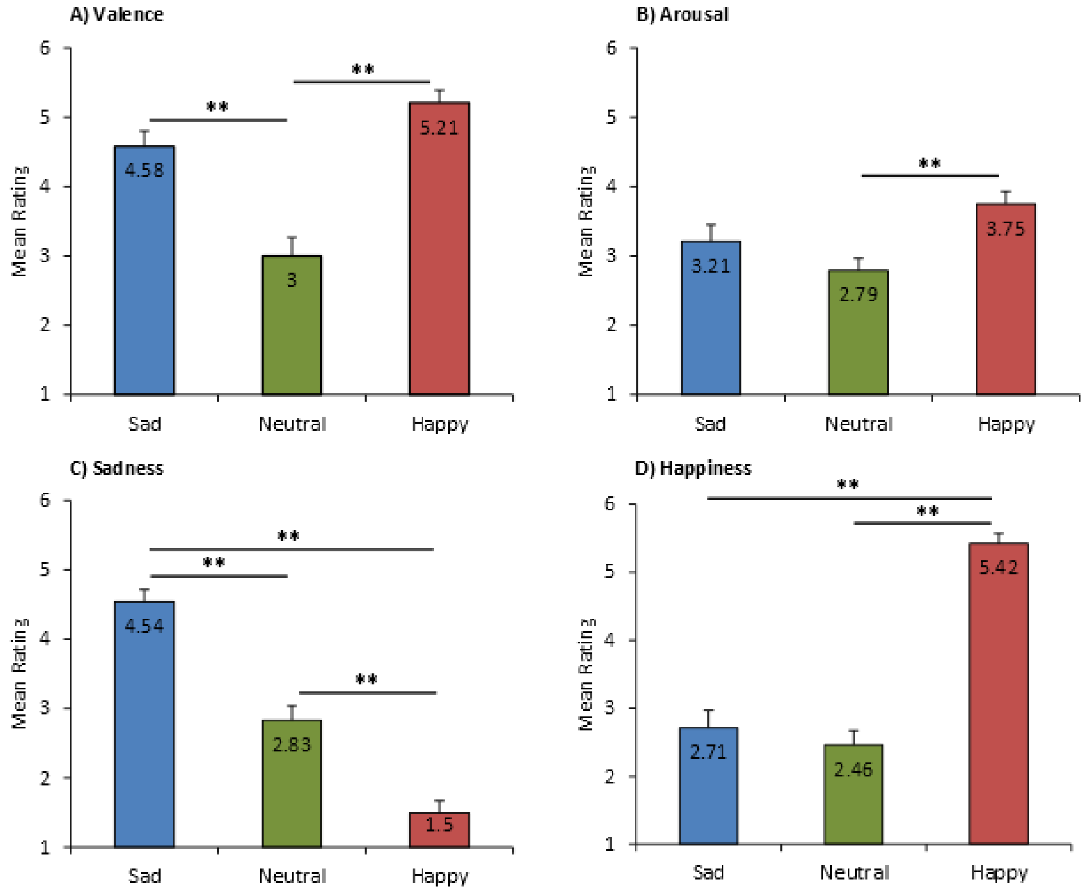

Figure S2. Emotions evoked by sad, happy, and neutral stimuli during the fMRI experiment. Participants rated their emotional state on four scales: valence, arousal, sadness, and happiness. Scales ranged from 1 (“very unpleasant”, “very calm”, “not at all”) to 6 (“very pleasant”, “very aroused”, “very much so”). Results were corrected for multiple comparisons. (**A**) Valence ratings did not significantly differ between sad and happy music, but were significantly lower during neutral compared with sad and happy music. (**B**) Arousal ratings did not differ significantly between sad and happy music as well as between sad and neutral music, but were significantly lower during neutral compared with happy music. (**C**) Sadness ratings were significantly higher during sad compared with happy and neutral music, as well as during neutral compared with happy

music. (**D**) Happiness ratings were significantly higher during happy compared with sad and neutral music, but did not differ significantly between sad and neutral music. Error bars indicate 1 *SEM*. ** $P < .001$.

Supplementary Tables

Table S1. Items Used in Experiment 1A

Thought probe	Question
Mind-wandering	
Mind-wandering	Where was your attention just before the music stopped?
Meta-awareness	How aware were you of where your attention was focused?
Content of thought	
Open-ended format	What were you thinking about just before the music stopped?
Valence	Was the content of your thoughts positive or negative?
Past	Were you thinking about something from the past?
Future	Were you thinking about something from the future?
Self-referentiality	To what extent were these thoughts about yourself?
Familiar people	Please indicate the extent to which your thoughts were about … people you know (e.g., family, friends, partner)
Unknown people	… unknown people
Movements	… body movements or dancing
Bodily sensations	… bodily sensations (e.g., feeling hot/cold/tired/hungry, smiling/frowning)
Musical structure	… thinking about the music (e.g., its melody, beat, harmony)
Evaluating the music	… evaluating the music (e.g., I like it because..)
Experiment	… thinking about the experiment
Form of thought	
Visual imagery	Did you think in images?
Inner language	Did you think in words?

Answers were given on scales from 1 (“not at all”) to 7 (“very much so”), except for mind-wandering (1 = “completely on the music” and 7 = “completely on something else”), meta-awareness (1 = “completely unaware” and 7 = “completely aware”), valence (1 = “very negative” and 7 = “very positive”), and the open-ended question.

Table S2. Percentages of Total Positive and Negative Emotion Words across Reports of Thoughts' Content during Sad and Happy Music

	Emotion words	
	Positive	Negative
Sad music	2.64%	2.77%
Happy music	3.67%	0.82%

Reports of participants' thoughts were analyzed with the LIWC software, which provided percentage of word usage over the pre-chosen categories of positive and negative emotion words.

Table S3. Emotion Ratings (Answer Scales 1-7) during the Slow (Sad and Happy) and Fast (Sad and Happy) Music Pieces Used in Experiment 1B

	slow tempo mean (SD)		paired *t*-test	fast tempo mean (SD)		paired *t*-test
	sad	happy	*P*-value	sad	happy	*P*-value
valence	4.93 (1.26)	4.93 (1.15)	0.96	5.03 (1.07)	5.00 (1.05)	0.82
arousal	2.80 (1.12)	4.39 (1.09)	< 0.001	3.25 (1.05)	5.09 (1.06)	< 0.001
sadness	4.91 (1.27)	2.04 (0.95)	< 0.001	4.40 (1.14)	1.91 (0.94)	< 0.001
happiness	2.61 (1.15)	4.91 (1.07)	< 0.001	3.13 (1.13)	5.11 (1.05)	< 0.001

Table S4. Results of the ECM Contrasts *Sad* > *Happy* and *Happy* > *Sad*, Corrected for Multiple Comparisons ($P < .05$)

**anatomical location**	**MNI coordinates**	**cluster size (mm³)**	***z*-value: max (mean)**
sad > happy			
vmPFC	-6 39 -8	1755	4.24 (3.22)
r cingulate gyrus/vPFC	15 39 -2	810	4.04 (3.03)
dmPFC	0 45 31	864	3.70 (3.13)
PCC	0 -36 52	378	3.22 (3.00)
PCC/PCu	21 -51 16	3699	4.18 (3.12)
l pIPL	-48 -69 43	864	3.31 (2.97)
r pIPL	54 -63 28	729	3.42 (3.01)
happy > sad			
r IFG (pars opercularis)	48 12 25	486	3.68 (3.21)

The outermost right column shows the maximal *z*-value within a cluster (with the mean *z*-value of all voxels within a cluster in parentheses). dmPFC = dorsomedial prefrontal cortex; IFG = inferior frontal gyrus; PCC = posterior cingulate cortex; PCu = precuneus; pIPL = posterior inferior parietal lobule; vmPFC = ventromedial prefrontal cortex; vPFC = ventral prefrontal cortex.

Table S5. Music Stimuli from Experiment 1A

Title	Artist/Composer	Album	Year	Genre	Duration (m)
Sad pieces					
Song for Bob	Nick Cave & Warren Ellis	The Assassination of Jesse James by the Coward Robert Ford	2007	soundtrack	2.14
Darcy's Letter	Dario Marianelli	Pride & Prejudice	2005	soundtrack, classical	2.04
Elo Hi	Goran Bregović	Queen Margot	1994	soundtrack	1.20
Xibalba	Clint Mansell	The Fountain	2006	soundtrack, contemporary classical	1.48
Happy pieces					
Papaya	Stelvio Cipriani	The Police Can't Move	1975	soundtrack	2.29
Grand Hotel Fox	Nicola Piovani	Life is Beautiful	1997	soundtrack	1.54
String Quartet in F major, Op. 77 No. 2 (Finale Vivace Assai)	Joseph Haydn	Haydn: String Quartets	1994	classical	1.29
What Players Are They	Patrick Doyle	Hamlet	1996	soundtrack, classical	1.34

Table S6. Music Stimuli from Experiment 1B

Title	Artist/Composer	Album	Year	Genre	Duration (s)
Slow pieces					
Sad					
The Black Dog and The Scottish Play	Hilmar Örn Hilmarsson	Angels of the Universe	2001	soundtrack	75
Interlude	Dirk Reichardt, Stefan Hansen, Max Berghaus	Barefoot	2005	soundtrack	45
The Threat of War	Alexandre Desplat	The King's Speech	2010	soundtrack, classical	44
Happy					
What Players Are They	Patrick Doyle	Hamlet	1996	soundtrack, classical	75
The Peter Pan Overture	Jan Kaczmarek	Finding Neverland	2004	soundtrack	45
Papaya	Stelvio Cipriani	The Police Can't Move	1975	soundtrack	44
Fast pieces					
Sad					
Monday	Ludovico Einaudi	Divenire	2006	classical	84
Death is The Road to Awe	Clint Mansell	The Fountain	2006	soundtrack, contemporary classical	35
The Spin	Greg Haines	Moments Eluding	2012	contemporay classical, ambient	45
Happy					
Wedding - Čoček	Goran Bregović	Underground	2000	soundtrack, balkan	84
Two Hornpipes (Tortuga)	Hans Zimmer	Pirates of the Caribbean: Dead Man's Chest	2006	soundtrack, classical	35
String Quartet in F major, Op. 77 No. 2 (Finale Vivace Assai)	Joseph Haydn	Haydn: String Quartets	1994	classical	45

Table S7. Music Stimuli from Experiment 2

Title	Artist/Composer	Album	Year	Genre	Duration (s)
Sad pieces					
Hamlet	Ennio Morricone	Hamlet	1990	soundtrack, classical	90
Sadness	Dirk Reichardt, Stefan Hansen, Max Berghaus	Barefoot	2005	soundtrack	78
The Imperfect Enjoyment	Michael Nyman	The Libertine	2005	soundtrack, contemporary classical	37
Death is The Road to Awe	Clint Mansell	The Fountain	2006	soundtrack, contemporary classical	35
Happy pieces					
À La Folie	Michael Nyman	6 Days, 6 Nights	1994	soundtrack, contemporary classical	90
Willoughby	Patrick Doyle	Sense and Sensibility	1995	soundtrack, classical	78
Meryton Townhall	Dario Marianelli	Pride & Prejudice	2005	soundtrack, classical	37
Two Hornpipes (Tortuga)	Hans Zimmer	Pirates of the Caribbean: Dead Man's Chest	2006	soundtrack, classical	35

References

Bagby, R.M., Parker, J.D., Taylor, G.J. (1994). The twenty-item Toronto Alexithymia Scale—I. Item selection and cross-validation of the factor structure. *Journal of Psychosomatic Research*, **38**, 23-32.

Eerola, T., Toiviainen, P. (2004). MIR in matlab: The MIDI toolbox. *Proceedings of the International Conference on Music Information Retrieval*, Barcelona, Spain. p. 22–7.

Kane, M.J., Brown, L.H., McVay, J.C., Silvia, P.J., Myin-Germeys, I., Kwapil, T.R. (2007). For whom the mind wanders, and when: An experience-sampling study of working memory and executive control in daily life. *Psychological Science*, **18**, 614-21.

Koelsch, S. (2014). Brain correlates of music-evoked emotions. *Nature Reviews Neuroscience*, **15**, 170-80.

Maess, B., Koelsch, S., Gunter, T.C., Friederici, A.D. (2001). Musical syntax is processed in Broca's area: An MEG study. *Nature Neuroscience*, **4**, 540-5.

Mas-Herrero, E., Marco-Pallares, J., Lorenzo-Seva, U., Zatorre, R.J., Rodriguez-Fornells, A. (2013). Individual differences in music reward experiences. *Music Perception*, **31**, 118-38.

Pehrs, C., Deserno, L., Bakels, J.H., *et al.* (2014). How music alters a kiss: Superior temporal gyrus controls fusiform-amygdalar effective connectivity. *Social Cognitive and Affective Neuroscience*, **9**, 1770-8.

Pereira, C.S., Teixeira, J., Figueiredo, P., Xavier, J., Castro, S.L., Brattico, E. (2011). Music and emotions in the brain: Familiarity matters. *PLoS ONE*, **6**, e27241.

Rush, A.J., Trivedi, M.H., Ibrahim, H.M., *et al.* (2003). The 16-item quick inventory of depressive symptomatology (QIDS), clinician rating (QIDS-C), and self-report (QIDS-SR): A psychometric evaluation in patients with chronic major depression. *Biological Psychiatry*, **54**, 573-83.
